# Intrahepatic bile duct mixed adenoneuroendocrine carcinoma: a case report and review of the literature

**DOI:** 10.1186/s13000-015-0439-1

**Published:** 2015-11-20

**Authors:** Sean L. Zheng, Vincent S. Yip, Federica Pedica, Andreas Prachalias, Alberto Quaglia

**Affiliations:** Department of Hepato-Pancreato-Biliary Surgery, King’s College Hospital, Denmark Hill, London, SE5 9RS UK; Liver Histopathology, Institute of Liver Studies, King’s College Hospital, Denmark Hill, London, SE5 9RS UK

**Keywords:** Neuroendocrine tumour, Mixed adeno-neuroendocrine carcinoma, Biliary tract

## Abstract

**Background:**

Mixed adeno-neuroendocrine carcinoma (MANEC) of the biliary tract is rare with only a few reported cases. Consequently, knowledge about their pathogenesis, histopathological characteristics and outcomes is sparce.

**Case presentation:**

A 53-year old man presented with epigastric pain on a background of excessive alcohol consumption. Contrast-enhanced computed tomography imaging of the liver revealed a central enhancing mass located at the bifurcation of right anterior and posterior portal veins. Magnetic resonance imaging demonstrated intrahepatic biliary duct dilatation distal to the mass. The patient underwent a right lobe hepatectomy and excision of the extrahepatic biliary tree with formation of a hepaticojejunostomy. Histopathological finding of the specimen revealed an intraductal tumour with predominant neuroendocrine immunohistochemical phenotype and infiltration into nearby tissue. An element of glandular differentiation on immunohistochemistry confirmed the lesion as MANEC.

**Conclusions:**

We present the first reported histopathological case of a MANEC arising from the intrahepatic bile ducts. This report aims to review what is known about primary neuroendocrine and mixed adeno-neuroendocrine carcinoma of the bile ducts, particularly in comparison to other types of biliary and hepatic tumours.

## Background

Malignancies affecting the extrahepatic bile duct are rare, accounting for around 0.1–0.2 % of all cancer diagnoses [1]. Of these cholangiocarcinoma is the commonest cause, with adenocarcinoma making up around 80 % of cases and other subtypes occurring much less frequently [2]. Rarely biliary ductal tumours with neuroendocrine differentiation arise within the extrahepatic bile ducts [3, 4]. Initial presentation may be associated with features of local disease (biliary colic, painless jaundice) or, occasionally, as a result of production of active hormones.

According to the latest World Health Organisation (WHO) classification system [5] biliary tract neuroendocrine tumours (BNET) are classified into neuroendocrine tumours (NET), neuroendocrine carcinomas (NEC), and mixed adeno-neuroendocrine carcinomas (MANEC). MANEC is a subtype that shows characteristics of both glandular and endocrine differentiation. MANEC of the biliary ductal system are extremely rare, with only a handful of published cases to-date [6, 7]. To date there have been no published cases of MANEC derived from the intrahepatic biliary ductal system. As a result little is known about their pathogenesis and outcomes.

We report a case of MANEC of the intrahepatic bile duct. Our aims are to describe its clinical presentation, and the detailed immunophenotypic characteristics of this tumour. Despite the paucity of cases in the literature, another objective is to compare the clinicopathologic characteristics of various biliary tumours, and predict outcomes of MANEC in biliary tumours.

## Case presentation

### Case history

A 53-year old man presented to his local district general hospital with epigastric pain. Relevant past medical history includes type 2 diabetes mellitus, and a history of excessive alcohol consumption of 44 units/week.

**Fig. 1:**
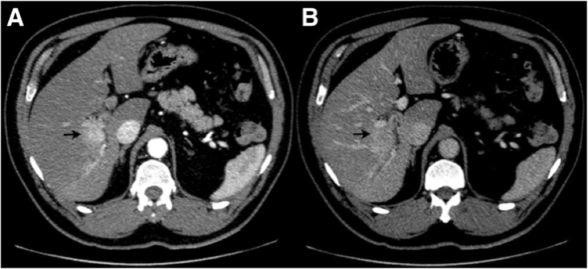
Dual phase abdominal contrast CT scan. Tumour at the bifurcation of the right anterior and posterior portal vein (*arrow*). Enhancement of lesion during arterial phase (**a**), with mild contrast washout in portal venous phase (**b**). Liver shows background steatosis

**Fig. 2:**
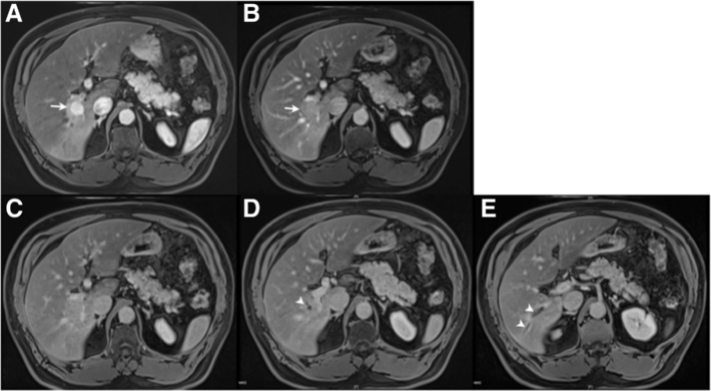
MRCP. Arterial phase demonstrating enhancement of tumour (*arrow*) (**a**), with images at 120 s post-contrast demonstrating washout of contrast (*arrow*) (**b**), and successive axial images at 10 min post-contrast showing dilatation of interlobular bile ducts (*arrowhead*) (**c**, **d**, **e**)

The patient underwent an abdominal dual-phase contrasted computed tomography (CT) scan. In addition to background liver steatosis and right liver atrophy, a central mass was identified at the bifurcation of the right anterior and posterior portal vein. The lesion was enhanced during the arterial phase, and mild contrast washout at the portal venous phase, suggestive of possible hepatocellular carcinoma (HCC) (Fig. 1a and b). The mass had increased in size as compared to previous imaging. Subsequent magnetic resonance imaging (MRI) liver confirmed the same mass in the right lobe with peripheral biliary ductal dilatation. This then raised the possibility of a clinical diagnosis of intrahepatic cholangiocarcinoma (Fig. 2a–e).

Preoperative bilirubin was 15 μmol/L (normal range: 4–22 μmol/L), alkaline phosphatase was 110U/L (42–98U/L) and aspartate transaminase was 39 IU/L (6–34 IU/L). Alpha-Feto Protein (AFP) and carbohydrate antigen 19–9 (CA19-9) were 3.7kU/ml (<4kU/ml) and 7 (<33kU/L) respectively.

This patient was then referred to our tertiary hepato-pancreato-biliary centre for further management. Having discussed this case in our multi-disciplinary meeting, fluorodeoxyglucose—positron emission tomography (FDG-PET) CT scan was performed and excluded extrahepatic metastasis. No FDG uptake was demonstrated within the lesion as compared to the background liver parenchyma.

The patient was listed for surgical resection with curative intent. Right lobe hepatectomy and excision of the extrahepatic biliary tree with the reconstruction of a hepaticojejunostomy was performed. There were no postoperative complications. Patient made a good post-operative recovery and was subsequently discharged home. 68Ga-DOTA-conjugated peptide PET scan completed 4 weeks post-operation did not demonstrate any evidence of residual disease.

### Histology and immunohistochemistry

A 20 mm diameter friable, greyish and pale tumor occupied the lumen of the right hepatic duct and infiltrated into the surrounding liver and hilar adipose tissue. The background liver appeared yellowish with vague accentuation of the lobular architecture.

**Fig. 3:**
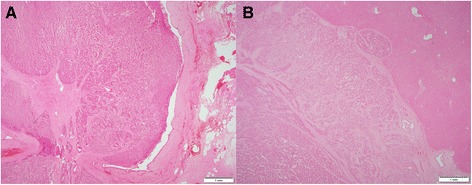
Low magnification view of intraductal (**a**) and periductal invasive component (**b**). H&E 20×

**Fig. 4:**
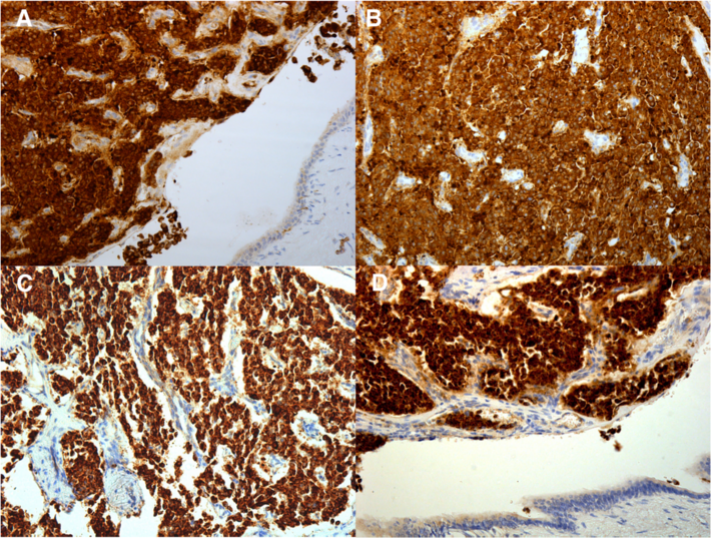
Intraductal component showing strong and diffuse staining for synaptophysin (**a** and **b**) and chromogranin (**c** and **d**)

**Fig. 5:**
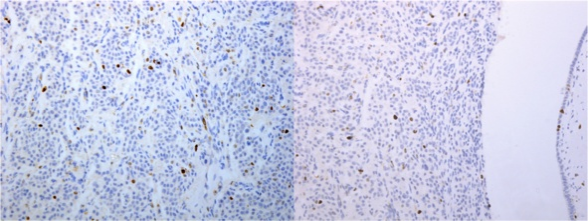
Ki67 analysis estimated to be up to 8 % with up to three mitotic figures present in 50 HPF

At light macroscopy (Fig. 3a and b), approximately half of the tumour mass formed an intraductal growth, with the rest of the tumour infiltrating into periductal fibrovascular tissue and local liver parenchyma. Both the intraductal and periductal infiltrative aspects were composed of large sheets of relatively monomorphic cells mixed with a rhabdoid or plasmacytoid appearance. Nuclei were generally round and regular and often showed a “salt-and-pepper” appearance with focally increased and dense chromatin. Immunohistochemistry in this cell population stained strongly and diffusely for synaptophysin (DAKO, M0776, 1:100) (Fig. 4a–b) and chromogranin (DAKO, 1:100) (Fig. 4c–d). The proliferative rate estimated with immunohistochemical analysis for Ki67 (MIB-1, DAKO, M0701, 1:100) was up to 8 % and up to three mitotic figures were present in 50 HPF (Fig. 5).

**Fig. 6:**
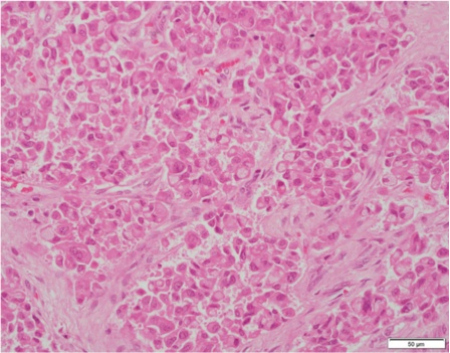
High magnification view of part of the lesion showing clarification of the cytoplasm and signet ring morphology. H&E 400×

**Fig. 7:**
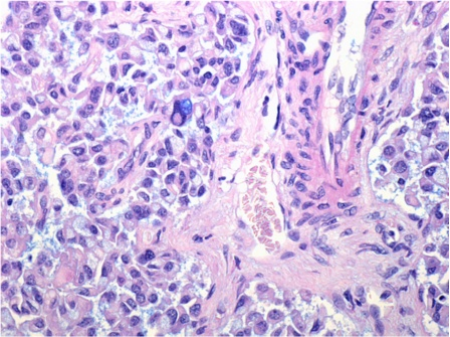
Alcian blue diastase PAS. Tumour cells with signet ring morphology showing weak cytoplasmic staining. 400×

**Fig. 8:**
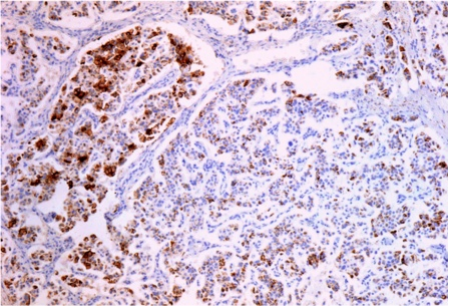
Numerous tumour cells in the areas showing signet ring morphology stain for MUC-1. 30×

**Fig. 9:**
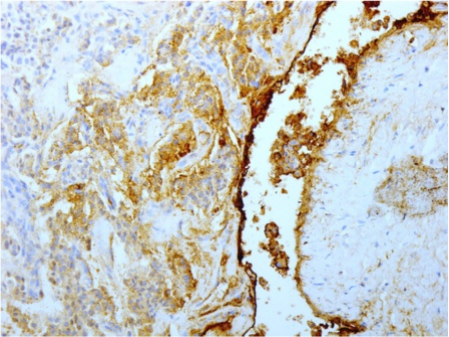
CA19.9 staining highlights the superficial component of the intraductal-growing tumor, most likely residual biliary epithelium

**Fig. 10:**
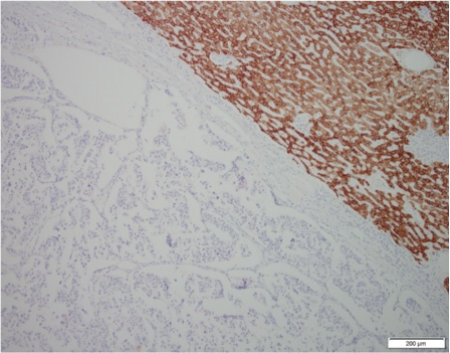
Hep-Par1 staining showing negative hepatocellular tumour differentiation, with normal adjacent liver parenchymal staining (*top right*)

A minor component of the cell population (estimated to be around 30 %) showed a signet ring appearance with clarification of the cytoplasm suggestive of intracellular mucin (Fig. 6), which stained weakly with alcian-blue-dPAS and showed focal acinar formation with intraluminal mucin secretion (Fig. 7). With the limitation of single epitope immunohistochemistry on serial sections this cell population appeared to retain the expression of chromogranin and synaptophysin. This also showed cytoplasmic or membranous expression of MUC-1, (Fig. 8) (Abcam, Ab696-250, 1:100) predominantly in the deeper aspect of the tumour, but did not stain for MUC-2 (Novocastra, NCL MUC2, 1:100), MUC5 (Novocastra, NCL MUC5, 1:100) or MUC-6 (Novocastra, NCL MUC6, 1:100). There was weak and diffuse nuclear staining for CDX2 (Leica Bond RTU, PA0535) throughout the tumour. Staining for CA19.9 (Leica RTU, PA 0424) highlighted the superficial component of the intraductal growing tumor, in keeping with residual biliary epithelium (Fig. 9). The background biliary epithelium did not reveal dysplasia or metaplastic changes. Hep-Par1 staining of tumour was negative (Fig. 10).

The background liver showed steatohepatitis, mainly macrovesicular, affecting around 30 % of the hepatocytes associated with a mild bridging fibrosis.

## Conclusions

We present a rare case of intrahepatic biliary MANEC and highlight the difficulty in establishing a pre-operative diagnosis [8]. The differential diagnoses in this case include intrahepatic cholangiocarcinoma, hepatocellular carcinoma and intrahepatic neuroendocrine carcinoma (Table 1 for clinicopathological comparison).

**Table 1:** Comparison of clinical, pathological and histological features of intrahepatic carcinomas

	Hepatocellular carcinoma	Cholangiocarcinoma	Biliary neuroendocrine carcinoma	Mixed adeno-neuroendocrine carcinoma
Common site	Liver parenchyma	Perihilar and extrahepatic, rarely intrahepatic [9, 30]	Extrahepatic biliary tract	Very rare, perihilar and extrahepatic bile ducts [6]
				No previous reports of intrahepatic MANEC
Laboratory abnormalities	Raised hepatitic enzymes	Intrahepatic—typically raised ALP and GGT, normal or mildly elevated bilirubin	Raised ALP and GGT	As cholangiocarcinoma and BNET
	Tumour markers—AFP [31]	Tumour markers CA19-9 and CEA may be raised, but lack sensitivity and specificity [12–14]		
Radiological features	Hyper-attenuating in arterial phase with portal venous phase washout on dual-phase contrast CT	Hypo or iso-attentuating compared to background liver on dual-phase contrast CT in both venous and arterial phases	Similar radiological findings to cholangiocarcinoma	As cholangiocarcinoma and BNET
	Increased T2 intensity on MRI	Evidence of biliary tract obstruction with proximal dilatation		
	Invasion of portal vein			
Histological features	Neoplastic cells resembling hepatocytes. It can have different type of architecture such as trabecular, acinar, pseudoglandular, compact and scirrhous	Invasive adenocarcinoma with variable sized tubular structures, formation of acini or micropapillary structures. The intraductal growth in the extrahepatic biliary tree can present as BilIN or IPNB [32]. It does not express chromogranin and synaptophysin extensively	These tumours are composed of cells superimposable to those of gut and pancreas endocrine cell and show diffuse positivity for Chromogranin A and synaptophysin without any other differentiation [5]	Adenocarcinoma component typically on tumour surface, with stromal, vascular and local lymph node invasion involving neuroendocrine components [6]
	Positive Hep-Par1 staining [28]			Neuroendocrine component usually shows higher proliferative activity [6]
Prognosis	Highly variable dependent on staging, grading, presence or absence of cirrhosis [33]	Better outcomes in intrahepatic tumours For R0-resected intrahepatic tumours—median survival 80 months, 5-year survival 63 % [9]	Dependent on grade (mitotic index and Ki-67 proliferation index) [7]	Dependent on proliferative activity of neuroendocrine component [6]
			Appears to have better long-term survival rate compared with other biliary tract malignancies [34]	45 month survival in one case [29]

Cholangiocarcinoma occur most frequently in the hilar region, at the confluence of the right and left hepatic ducts. Those involving only the intrahepatic ducts are less frequent, 8 % of all cholangiocarcinomas in one large retrospective study [9]. The pattern of tumour growth and its anatomical location mean that obstructive jaundice is a late feature and tumour size tends to be larger with local infiltration, as occurred in our case [9–11]. Blood or bile testing for tumour markers such as CA19-9 and carcinoembryonic antigen (CEA), are often of limited diagnostic utility in isolation as both markers lack sensitivity and specificity, as was evident in this case [12–14].

Mass-forming intrahepatic cholangiocarcinoma may be difficult to differentiate from HCC on imaging. Dual-phase contrasted CT is frequently a useful tool in differentiating these pathologies with their characteristic radiological findings. Cholangiocarcinomas are typically hypo- or iso-attenuating relative to normal liver parenchyma at both arterial and portal venous phases, with enhancement only in the delayed phase [8, 15]. This finding reflects the hypovascular desmoplastic composition of cholangiocarcinoma. HCC on the other hand are more hypervascular and tend to show arterial phase enhancement with portal venous phase washout. The lesion in this case was unusual as contrast CT imaging showed arterial enhancement with mild washout at portal venous phase suggestive of HCC, yet the MRI liver demonstrated peripheral biliary obstruction, which is more in keeping with a bile duct neoplasm. One large retrospective study demonstrated that a significant proportion of mass-forming intrahepatic cholangiocarcinomas had arterial enhancement on dual-phase CT [16].

BNET are very rare and represent less than 0.3 % of all NET [3, 7]. They were first described in 1959, with only 150 cases published since then. The majority of these tumours are located in the extrahepatic biliary tracts, with smaller numbers originating at the bifurcation of the extrahepatic biliary tree [3, 7, 17]. In clinical practice, these lesions are difficult to diagnose and to distinguish from cholangiocarcinoma preoperatively. Certain clinical features can be useful in distinguishing one from the others. For instance, BNET tend to affect younger patients, whilst aggressive local invasion and distant metastatic disease is seen more frequently in adenocarcinoma. A pre-operative diagnosis can be made by examining brush cytology [18] or detecting raised serum tumour markers [7], though these methods have high false negative rates. A more precise pre-operative diagnostic technique is the use of spyglass endoscopy to obtain tissue biopsy from the lesion of the biliary ductal system, though availability of this investigative modality is often limited. Most of the time, surgical resection is the treatment of choice in the first instance, particularly when the lesion is deemed resectable on imaging, with a final diagnosis made following histopathological examination of the resected specimen. Pure BNET show typical histological features of NET and may show production of hormones, including somatostatin, serotonin and gastrin [7]. Somatostatin analogues such as octreotide, have been shown in clinical trials to be efficacious in improving symptoms and reducing tumour growth [19]. Prognostic data for BNET are limited due to their rarity. According to the most recent WHO classification, they should be graded as conventional NET taking into account in particular mitotic activity and Ki67 proliferative index [5, 20]: G1: mitotic count <2 HPF and/or Ki67 index <2 %; G2: mitotic count 2–20 per 10 HPF and/or Ki67 index 3–20 % and G3: mitotic count >20 per 10 HPF and/or Ki67 index >20 %. Overall outcomes appear to be better than in other types of biliary tract malignancies.

Our case differs from conventional BNET and cholangiocarcinoma due to the mixed but tightly intermingled neoplastic components on histological examination. The bulk of the mass is represented by a moderately differentiated NET, which stains diffusely for chromogranin and synaptophysin. A minor cell population scattered throughout the tumor and more prominently in its deep aspect closer to the nearby liver parenchyma is composed of cells with signet ring morphology. These cells highlight well with Alcian blue/periodic acid-Schiff (ALCIAN-PAS) and MUC1 staining, demonstrating acid mucus production by these cells and providing support to divergent glandular differentiation. This dual phenotype fits with the category of biliary MANEC and resembles the case described by Harada [6] with the adenocarcinomatous component found at the peripheral.

Biliary MANEC is exceedingly rare with only a few cases published in the literature [21–26]. In one series of hepatobiliary MANEC, only 9 cases were identified as MANEC [6]. Two of these cases were categorised as hepatic hilar MANEC, with the rest originating from gallbladder and extrahepatic bile ducts. To date and to our knowledge there have been no published cases of MANEC originating from intrahepatic bile ducts. Biliary MANEC have a phenotype that morphologically resembles both adenocarcinoma and NEC. According to the updated WHO classification, it was suggested that to be classified as MANEC, at least 30 % of the main lesion excluding any broad invasive area had to be made up of each component [27]. Harada et al. found that the adenocarcinoma components were predominantly located on the surface of the tumours, whilst the neuroendocrine components were found deeper and associated more often with stromal, vascular and lymphatic invasion [6]. Our case is similar because the glandular component was more pronounced at the peripheral intraductal part of the tumour; whereas the infiltrating component was mainly moderately differentiated (G2) neuroendocrine (Ki67 up to 8 %). Nonetheless, the definition of malignancy in this peculiar category of tumours is still not well established [5].

As with Harada et al. [6], the proliferative index in our case was higher in the neuroendocrine component compared with the adenocarcinomatous population, suggesting that neuroendocrine is the population upon which prognosis depends.

Additional histopathological diagnoses to be considered in mixed differentiated intrahepatic tumours include intraductal papillary neoplasms (IPMN) and HCC with neuroendocrine differentiation. IPMN are a precursor lesion that may rarely show neuroendocrine differentiation [7], but are characterized by protruding papillae covered by dysplastic epithelium with mucin secretion. In our case, there was an abrupt passage from the normal bile duct epithelium to the neoplastic mass without any evidence of preneoplastic lesion. Moreover, the epithelial marker CA19.9 and MUC1 stained only part of the tumour suggesting that there are two separate and discrete albeit mixed components. MANEC is therefore more likely to be derived from the adenocarcinomatous cells, rather than from neuroendocrine populations [21]. HCC with neuroendocrine differentiation has been anecdotally reported and remains unrecognised in the most recent WHO classification system. Our tumour did not resemble HCC morphologically, and Hep-Par1 staining was negative [28].

With only a paucity of cases, it is difficult to fully ascertain the long-term prognosis of biliary MANEC. There had been a report of 45-month survival in one patient who underwent surgical treatment alone [29]. However, that patient had a composite adenocarcinoma (40 %) and NEC (60 %) of the common bile duct. Although the location of the tumour is distinguishably different from our case, both cases had low proliferative fractions (<10 % Ki-67-positive tumour cells), which have been shown to provide significant prognostic information [20].

In summary, we report an intraductal invasive neoplasm with divergent predominantly neuroendocrine differentiation arising from the right intrahepatic biliary duct in keeping with a biliary MANEC. To our knowledge, this is the first reported case of an intrahepatic biliary MANEC, with the few previously reported cases affecting the extrahepatic biliary ductal system. On-going research is necessary to increase our understanding of the pathogenesis of this rare disease.

## Consent

Written informed consent was obtained from the patient for publication of this Case Report and any accompanying images. A copy of the written consent is available for review by the Editor-in-Chief of this journal.

## Abbreviations

BNET: Biliary tract neuroendocrine tumours
NET: Neuroendocrine tumours
NEC: Neuroendocrine carcinomas
MANEC: Mixed adeno-neuroendocrine carcinomas
CT: Computed tomography
HCC: Hepatocellular carcinoma
MRI: Magnetic resonance imaging
AFP: Alpha-Feto Protein
CA19-9: Carbohydrate antigen 19–9
CEA: Carcinoembryonic antigen
FDG: Fluorodeoxyglucose
PET: Positron emission tomography
IPMN: Intraductal papillary neoplasms
